# Tumor suppressors inhibit reprogramming of African spiny mouse ( *Acomys*) fibroblasts to induced pluripotent stem cells

**DOI:** 10.12688/wellcomeopenres.18034.1

**Published:** 2022-08-18

**Authors:** Aaron Gabriel W. Sandoval, Malcolm Maden, Lawrence E. Bates, Jose C.R. Silva

**Affiliations:** 1 Wellcome-MRC Cambridge Stem Cell Institute, University of Cambridge, Cambridge, CB2 0AW, UK; 2 Department of Biochemistry, University of Cambridge, Cambridge, CB2 1GA, UK; 3 Department of Biology & UF Genetics Institute, University of Florida, Gainesville, FL, USA; 4 MRC Human Genetics Unit, Institute of Genetics and Cancer, University of Edinburgh, Edinburgh, EH4 2XU, UK; 5 Guangzhou Laboratory, Guangzhou International Bio Island, Guangzhou 510005, Guangdong Province, China

**Keywords:** African spiny mouse, Acomys, regeneration, reprogramming, induced pluripotent stem cell, dedifferentiation, SV40 Large T antigen, tumor suppressor

## Abstract

**Background: **The African spiny mouse ( *Acomys*) is an emerging mammalian model for scar-free regeneration, and further study of *Acomys *could advance the field of regenerative medicine. Isolation of pluripotent stem cells from *Acomys *would allow for development of transgenic or chimeric animals and *in vitro *study of regeneration; however, the reproductive biology of *Acomys *is not well characterized, complicating efforts to derive embryonic stem cells. Thus, we sought to generate *Acomys* induced pluripotent stem cells (iPSCs) by reprogramming somatic cells back to pluripotency.

**Methods: **To generate *Acomys* iPSCs, we attempted to adapt established protocols developed in *Mus*. We utilized a PiggyBac transposon system to genetically modify *Acomys *fibroblasts to overexpress the Yamanaka reprogramming factors as well as mOrange fluorescent protein under the control of a doxycycline-inducible TetON operon system.

**Results: **Reprogramming factor overexpression caused *Acomys *fibroblasts to undergo apoptosis or senescence. When SV40 Large T antigen (SV40 LT) was added to the reprogramming cocktail, *Acomys *cells were able to dedifferentiate into pre-iPSCs. Although use of 2iL culture conditions induced formation of colonies resembling *Mus *PSCs, these *Acomys *iPS-like cells lacked pluripotency marker expression and failed to form embryoid bodies. An EOS-GiP system was unsuccessful in selecting for bona fide *Acomys *iPSCs; however, inclusion of Nanog in the reprogramming cocktail along with 5-azacytidine in the culture medium allowed for generation of *Acomys *iPSC-like cells with increased expression of several naïve pluripotency markers.

**Conclusions: **There are significant roadblocks to reprogramming *Acomys* cells, necessitating future studies to determine *Acomys*-specific reprogramming factor and/or culture condition requirements. The requirement for SV40 LT during *Acomys *dedifferentiation may suggest that tumor suppressor pathways play an important role in *Acomys *regeneration and that *Acomys *may possess unreported cancer resistance.

## Introduction

Typically development occurs in a unidirectional, irreversible manner; however, this process can be reversed, and differentiated cells can be returned to an early embryo-like pluripotent state through transcription factor overexpression ^1^. These induced pluripotent stem cells (iPSCs) recapitulate all characteristics of embryonic stem cells (ESCs) ^2^ and are believed to be essentially equivalent ^3^. Although naïve pluripotent stem cells have been derived from a few species ^4– 6^, the signal requirements to support this state are not well defined for most species. Thus, the emergence of iPSCs has provided an alternative way to acquire PSCs, and these cells can then be used to identify critical signaling requirements. In species where embryos are not easily accessible, reprogramming presents a more convenient method of PSC generation ^7^.

Since iPSCs retain characteristics of the species from which they are derived, they present useful *in vitro* models for biological phenomena ^8^. For instance, thirteen-lined ground squirrel *(Ictidomys)* iPSCs exhibit cold adaptation ^9^, while naked mole rat *(Heterocephalus)* iPSCs exhibit cancer resistance ^10– 12^. Another such trait of interest is regeneration, for which most adult animals, including *Mus* and humans, demonstrate a limited capacity. Though most models of regeneration are invertebrates or lower vertebrates ^13^, the African spiny mouse *(Acomys)* presents a unique mammalian model of multi-organ regeneration ^14^, and *Acomys* iPSCs may retain regenerative characteristics, enabling *in vitro* study of regeneration. Since the naïve pre-implantation epiblast is a stage of development exclusive to mammals, *Acomys* iPSCs would present the first and only PSCs from an organism both developmentally similar to humans and capable of such extensive regeneration.

Importantly, iPSCs could prove valuable in expanding the repertoire of tools to study *Acomys*. Since genetically modified iPSCs are capable of germline transmission, production of transgenic animals would be possible ^15^, allowing for the interrogation of individual gene functions in *Acomys* regeneration. iPSCs would also allow for the generation of *Acomys-Mus* interspecies chimeras ^16^, facilitating investigation of how cells from each species differentially contribute to wound healing. Furthermore, organoids generated through differentiation of iPSCs could allow for *in vitro* study of *Acomys* development and regeneration ^17^. Organoid models for hair-bearing skin are especially attractive given *Acomys’s* ability to regenerate skin ^18^, but organoids from tissues across the body could enable study of organs whose regenerative capacity is yet to be assessed in *Acomys*.

In this work, we attempt to adapt *Mus* reprogramming protocols for use in *Acomys*; however, due to the unique physiology of this regenerative rodent along with a lack of available research resources developed for use in this non-traditional model, we encounter several biological and technical roadblocks impeding the generation of *Acomys* iPSCs.

## Methods

### Cell lines

*Acomys* fibroblasts derived from the dorsal skin of newborn *Acomys* pups were obtained from our in-house colony at University of Florida.

*Acomys* reprogramming intermediates were generated by transfecting fibroblasts with pPBase, pPB-CAG-rtTA-IRES-bsd, pPB-TRE-MKOS-imO, pPB-CAG-SV40LT-PGK-hyg, pPB-EOS-GiP, and pPB-TRE-Nanog-PGK-hyg as indicated.

Media were supplemented with 1 μg/ml doxycycline (MP Biomedicals) and 1 μg/ml puromycin (Sigma Aldrich) as indicated.

### Cell culture

*Acomys* cells were cultured in serum-based MEF medium, KSR LIF, N2B27 2iL, N2B27 4iL, FAX, t2iL Gö XYaa, or PXGL as indicated on tissue culture plastic (Falcon) coated with 0.15% gelatin (Sigma Aldrich) in DPBS (Sigma Aldrich) as indicated at 37°C, 5% CO _2_, and 3% O _2_.

MEF medium was composed of GMEM without L-glutamine (Sigma Aldrich), 10% FCS (Labtech), 2mM L-glutamine (Gibco), 0.1mM 2-mercaptoethanol (Gibco), 1X MEM non-essential amino acids (Sigma Aldrich), 1mM Sodium Pyruvate (Sigma Aldrich), 1X penicillin-streptomycin (Sigma Aldrich), and 20 ng/ml mLIF (homemade: Department of Biochemistry).

KSR LIF was composed of GMEM without L-glutamine, 10% KOSR (Gibco), 1% FCS (Labtech), 2mM L-glutamine (Gibco), 0.1mM 2-mercaptoethanol (Gibco), 1X MEM non-essential amino acids (Sigma Aldrich), 1mM Sodium Pyruvate (Sigma Aldrich), 1X penicillin-streptomycin (Sigma Aldrich), and 20 ng/ml mLIF (homemade: Department of Biochemistry)

N2B27 was composed of Neurobasal (Gibco) and DMEM/F12 (Gibco) in a 1:1 ratio, 0.5% N2 (homemade: WT-MRC CSCI), 1% B27 (Gibco), 2 mM L-glutamine, 0.1 mM 2-mercaptoethanol, and 1X penicillin-streptomycin.

N2B27 2iL was composed of N2B27 supplemented with 3 μM CHIR99021 (Stewart lab, Dresden), 1 μM PD0325901 (Stewart lab, Dresden), and 20 ng/ml mLIF.

N2B27 4iL was composed of N2B27 2iL supplemented with 1 μM A83-01 (Tocris) and 0.1 μM PD173074 (Tocris).

FAX was composed of N2B27 supplemented with 12.5 ng/ml FGF2 (homemade: Department of Biochemistry, University of Cambridge), 20 ng/ml Activin A (homemade: Department of Biochemistry, University of Cambridge), and 2 μM XAV939 (Tocris).

T2iL Gö XYaa was composed of 1 µM CHIR99021, 1 µM PD0325901, 10 ng/ml mLIF, 2 µM Gö6983 (Tocris), 2 μM XAV939, 10μM Y-27632, 125 μM Ascorbic acid.

PXGL was composed of N2B27 supplemented with 1 μM PD0325901, 2 μM XAV939, 2 μM Gö6983, and 10 ng/mL mLIF.

Media were supplemented with 1 μg/ml doxycycline (MP Biomedicals), 0.5 or 1 μM 5-azacytidine (Sigma Aldrich), or 1 μg/ml puromycin (Sigma Aldrich) as indicated.

### Passaging and freezing cells

*Acomys* fibroblasts or reprogramming intermediates were passaged by dissociating with pre-warmed TrypLE Express (Gibco) or Accutase (Millipore), respectively, diluting 1:10 in DMEM/F12, pelleting by centrifugation at 300g for 3 minutes, aspirating supernatant, resuspending pellet, and plating cells.

Cells were frozen in N2B27 and DMSO (Applichem) in a 9:1 ratio at -80°C before transfer to liquid nitrogen for long-term storage.

### Fibroblast reprogramming

Effectene (Qiagen) was used for *Acomys* fibroblast transfections. One day prior to transfection, *Acomys* iPSCs were plated at 15,000 cells cm ^-2^ in a 6-well plate in MEF medium. On the day of transfection, 180 μl Buffer EC, 1 μg total of all piggyBac plasmids of interest, 0.2 μg PBase plasmid, and 9.6 μl Enhancer were combined and incubated at room temperature for 4 minutes. Then, 30 μl Effectene reagent was added and incubated at room temperature for 10 minutes. Medium was replaced with 1 ml fresh MEF medium. Following incubation, mixture was combined with 1 ml MEF medium and added dropwise to cells. Medium was replaced the following day with KSR LIF supplemented with 1 μg/ml doxycycline to induce reprogramming.

### Embryoid body (EB) differentiation

Three different EB differentiation protocols were attempted using *Acomys* iPS-like cells. Prior to each, *Acomys* iPS-like cells were prepared by dissociating with pre-warmed Accutase, diluting 1:10 in DMEM/F12, pelleting by centrifugation at 300g for 3 minutes, aspirating supernatant, resuspending pellet in DMEM/F12, pelleting by centrifugation at 300g for 3 minutes again, aspirating supernatant, and resuspending in MEF medium.

*Round Bottom Well:* Cells were diluted to 16,500 cells/ml, and 30 μl was pipetted into each well of a non-adherent 96-well round bottom plate. Empty wells were filled with DPBS to minimize evaporation. After 3 days, cells were transferred to a non-adherent 10 cm dish for suspension culture in MEF medium.

*Suspension Culture:* 1,500,000 cells were transferred to an uncoated, non-adherent for suspension culture in MEF medium.

*Hanging Drop:* Cells were diluted to 16,500 cells/ml or 33,000 cells/ml, and 30 μl was pipetted onto the lid of an uncoated, non-adherent 10 cm dish then inverted for hanging drop culture. The 10 cm dish was filled with DPBS to minimize evaporation. After 3 or 5 days, cells were transferred to a non-adherent 10 cm dish for further suspension culture in MEF medium.

### Plasmids and cloning

**Table 1.** Plasmids.

Plasmid Name	Source
pPBase (CMV-PBase)	Silva lab stocks
pPB-CAG-rtTA-IRES-puro	Silva lab stocks
pDONR211	Life Technologies
pPB-CAG-Dest-PGK-bsd	Silva lab stocks
pPB-TRE-MKOS-imO	Gift from Dr. Keisuke Kaji
pPB-CAG-rtTA-IRES-bsd	Silva lab stocks
pEntr-SV40LT	Thermo Fisher
pPB-CAG-Dest-PGK-hyg	Silva lab stocks
pPB-CAG-SV40LT-PGK-hyg	Generated through Gateway Cloning LR reaction (Thermo Fisher)
pPB-EOS-GiP	Silva lab stocks
pPB-TRE-Nanog-PGK-hyg	Silva lab stocks

### RNA extraction

RNeasy Mini Kit (Qiagen) was used to isolate RNA according to manufacturer instructions. Cells were harvested by aspirating medium and adding Buffer RLT for lysis. Cell lysate was transferred to QIAshredder columns for homogenization. Homogenized cell lysate was stored at -80°C until RNA extraction. During RNA extraction, on-column DNA digest with RNase-free DNase I was performed. RNA quantity and purity were assessed using a Nanodrop ND-1000 spectrophotometer.

### cDNA synthesis

SuperScript III First-Strand Synthesis SuperMix for RT-qPCR (Life Technologies) was used to reverse-transcribe RNA to cDNA. Quantities of RNA up to 1 μg were normalized across all samples of a particular experiment. cDNA was diluted with water to an approximate final concentration of 1 ng/μl.

### RT-qPCR

Fast SYBR Green Master Mix (Life Technologies) along with sample cDNA, and primers targeting both endogenous and exogenous expression of the genes listed in Table 2 were used to perform qPCR in technical triplicate reactions in an Applied Biosystems StepOne Real Time PCR system (Thermo Fisher). Default cycling parameters for SYBR Green regents were used (95°C hold for 20s, 40 cycles of 95°C for 3s, 60°C for 30s with data collection, then a melt curve was generated by a 15s hold at 95°C, 1 minute hold at 60°C, and gradual ramp up to 95°C with data collection).

**Table 2.** Acomys/Mus RT-qPCR primers.

Gene	Primer	Sequence
Pgk1	Fw Rv	GACTTGGTTCCCCTGGCAAA GGGCTTGGACTGTGGTACTG
Oct4	Fw Rv	TGTTCAGCCAGACCACCATC GCTTCCTCCACCCACTTCTC
Klf4	Fw Rv	TCTTCCCCTCTTTGGCTTGG GCCCAACTACCCTCCTTTCC
Gbx2	Fw Rv	CCAGGCAAATTGTCATCTGAGC AGACGAGTCAAAGGTGGAAGA
Tcfp2l1	Fw Rv	ACACCTTGATCTGGCAGCTG CTTTTCGGGTGCAGATTGACA
Tbx3	Fw Rv	TCGGAGCAGAGTTTGGGTG CTCGGTGGCTGTGGACTC
Fgf4	Fw Rv	GACCAGCCGCTTCTTCGTAG GTGTGCTTCCGAGGCTGAG

### Primer design

To analyze gene expression *,* we designed RT-qPCR primers in regions of the transcriptome shared between *Acomys* and *Mus* in order to verify proper primer binding and amplification using *Mus* ESCs as a positive control. We utilized a transcriptome assembled by sequencing early-stage *Acomys* embryos ^19^ to align *Mus* cDNA sequences with *Acomys* sequences. *Mus* sequences came from the Ensembl genome browser using the CL57BL6 reference strain.

### Data analysis

Representative microscope images are shown to illustrate qualitative changes in morphology and fluorescent protein expression. Brightness and contrast have been altered using Fiji ^20^ for the purpose of clarity. qPCR analyses of gene expression represent single experiments, and therefore have not been statistically analyzed.

## Results

### SV40 LT facilitates the early stages of Acomys reprogramming

**Figure 1.**
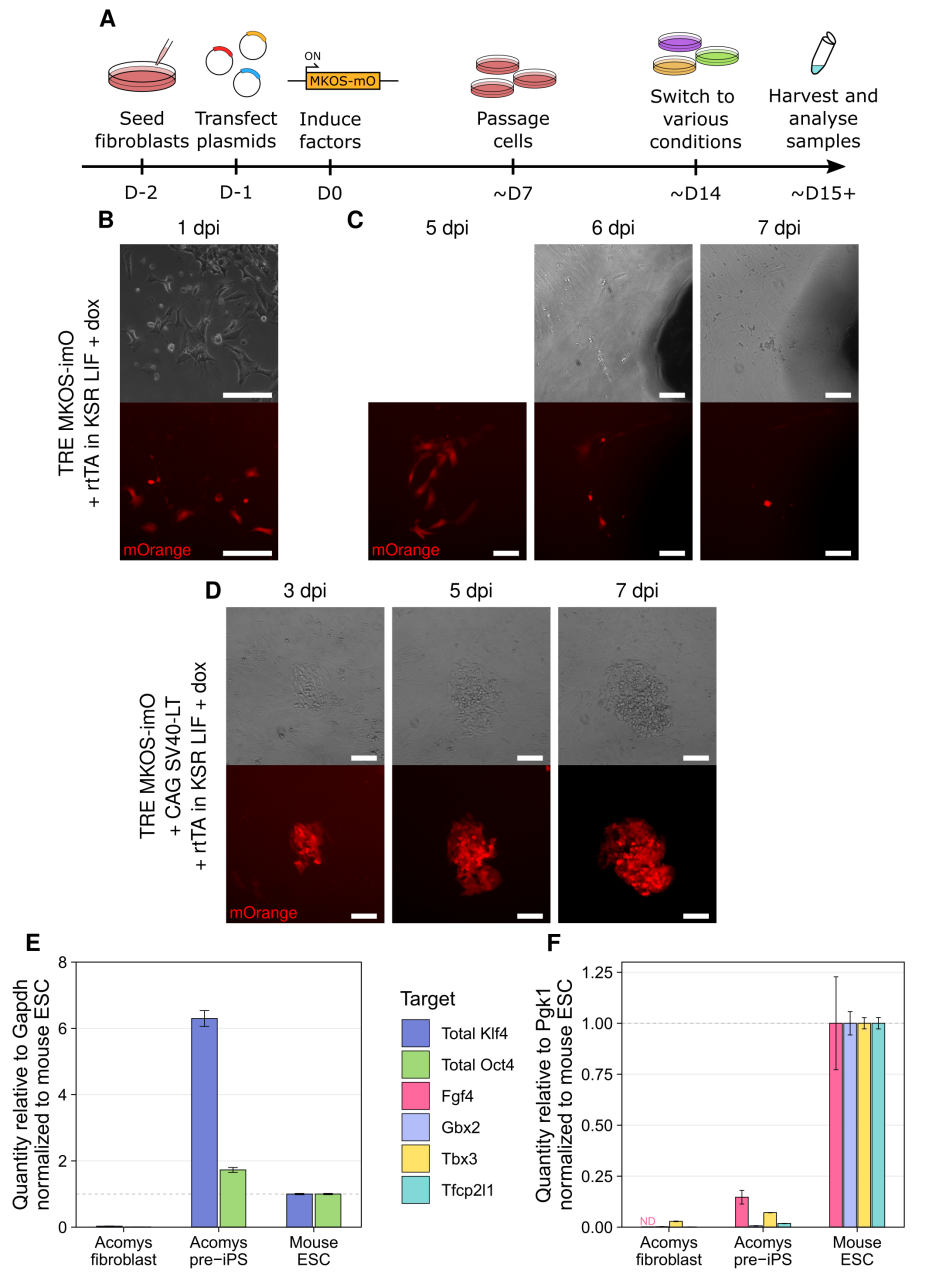
SV40 LT is required for Acomys fibroblasts to successfully dedifferentiate into pre-iPSCs. **A**) Schematic of proposed strategy for reprogramming *Acomys* fibroblasts to iPSCs based on protocols developed in *Mus.* **B**– **C**) Phase and mOrange images of reprogramming fibroblasts in KSR LIF dox at 1 dpi ( **B**) or followed from 5-8 dpi ( **C**). Scale bars represent 100 μm. **D**) Phase and mOrange images of reprogramming fibroblasts expressing SV40 LT in KSR LIF dox followed from 3-7 dpi. Scale bars represent 100 μm. E-F) RT-qPCR analysis of reprogramming factor ( **E**) and naïve pluripotency marker ( **F**) expression in *Acomys* pre-iPSCs, *Acomys* fibroblasts, and *Mus* ESCs. Mean expression is shown relative to the stated housekeeping gene and normalized to Mus ESC level, ± standard deviation (SD) (n=3 technical replicates). ND = not detected.

Initially, we attempted to reprogram *Acomys* fibroblasts to an iPSC identity through overexpression of the conventional Yamanaka factors, cMyc, Klf4, Oct4, and Sox2 (MKOS), combined with a media change to conditions supportive of naïve PSCs, as this is an effective protocol for the reprogramming of *Mus* fibroblasts ^21^ ( Figure 1A).

We transfected fibroblasts with a polycistronic cassette containing MKOS separated by self-cleaving 2A sequences along with an mOrange fluorescent protein connected via an IRES element (see Table 1 for plasmids), allowing for the expression of all 4 Yamanaka factors as well as mOrange under the control of a TetO promoter activated in the presence of doxycycline (dox) and rtTA ^22^. The reprogramming cassette was flanked by piggyBac arms, allowing for random integration into the genome ^23^. A constitutively expressed rtTA plasmid and non-integrating piggyBac transposase were also transfected.

At 1 day post-induction (dpi), several mOrange-fluorescent fibroblasts were present ( Figure 1B). While we initially observed proliferation of these cells, over longer time periods we found that this fluorescent population was lost either through transgene silencing or cell death. Following a group of fluorescent cells from 5 dpi to 8 dpi, it became clear that most cells expressing the reprogramming factors were dying, and specifically we observed widespread death around 6dpi ( Figure 1C). Few fluorescent cells remained after this period of cell death, and the surviving cells were non-proliferative, had not changed morphology, and were deemed senescent. Failure to induce dedifferentiation suggested there are roadblocks to reprogramming in *Acomys.*

Since c-Myc plays a role in both apoptotic signaling and cellular senescence ^24^, we reasoned this oncogene might be inhibiting reprogramming. Although reprogramming is possible without c-Myc in other species, the process is substantially delayed, efficiency is decreased, and germline transmissibility is compromised ^25^. Thus, we sought a way to overcome the negative effects of c-Myc while still including it in the reprogramming cocktail. SV40 large tumor antigen (SV40 LT) has previously been used to combat c-Myc-induced cellular toxicity and increase reprogramming efficiency by inhibiting the p53 and Rb tumor suppressor pathways ^10, 12, 26, 27^. We investigated whether SV40 LT could similarly abrogate the toxic effects of c-Myc in *Acomys*.

We added a piggyBac, constitutively expressed SV40 LT construct to the reprogramming cocktail. Compared to cells expressing MKOS alone, we observed more robust proliferation with the addition of SV40 LT. By 3 dpi, colonies containing morphologically distinct cells began to emerge ( Figure 1D). These continued to expand over time, not showing the gradual loss of mOrange signal that we observed in cells expressing MKOS alone. Drawing comparisons to Mus fibroblast reprogramming in which highly proliferative, yet incompletely reprogrammed, intermediates arise soon after overexpression of MKOS, we assumed that these cells were likely to be pre-iPS-like cells. In *Mus*, these often exhibit an ESC-like morphology and show partial upregulation of select pluripotency markers while downregulating somatic markers ^21^.

*Acomys* pre-iPSCs expressed total Oct4 and Klf4 exceeding *Mus* ESC levels, verifying reprogramming cassette expression ( Figure 1E). To assess pluripotency, we evaluated 4 well-characterized markers of *Mus* and human naïve pluripotency (Gbx2, Tcfp2l1, Tbx3, and Fgf4) using primers designed to amplify both *Mus* and *Acomys* transcripts (see Table 2 for primer details). Compared to fibroblasts, all 4 markers were upregulated to varying degrees in *Acomys* pre-iPSCs ( Figure 1F). Thus, overexpression of the Yamanaka factors combined with SV40 LT allows us to overcome the apoptosis and senescence caused by MKOS alone, permitting dedifferentiation of *Acomys* fibroblasts, and leads to slight upregulation of some components of the pluripotency network, as would be expected from pre-iPSCs ^21^.

### 2iL culture condition allows for conversion of Acomys pre-iPSCs to iPS-like colonies

**Figure 2.**
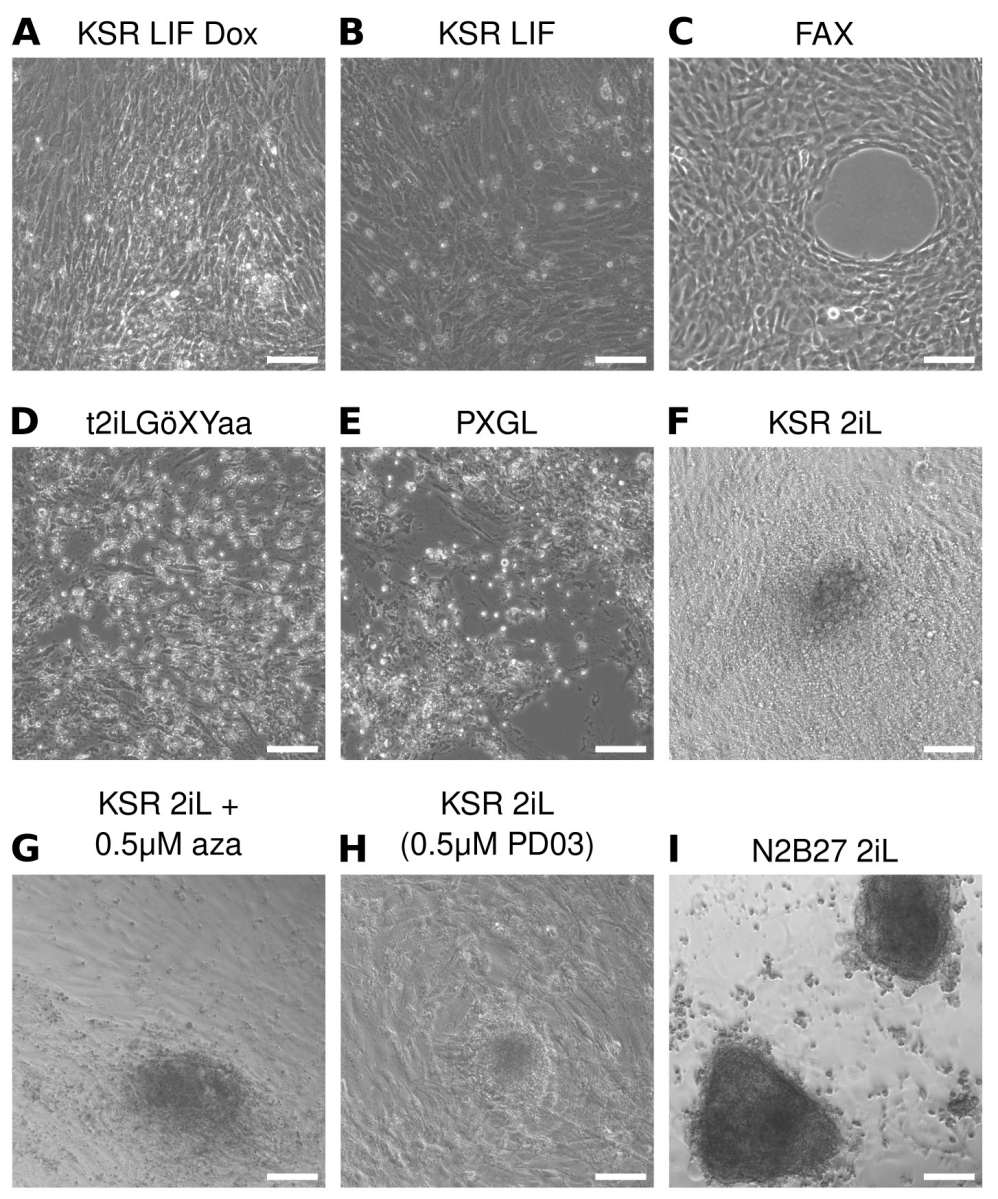
2iL culture conditions support formation of colonies. **A**– **I**) Phase images of pre-iPSCs 10 days after switching into KSR LIF dox ( **A**), KSR LIF ( **B**), FAX ( **C**), t2iL Gö XYaa ( **D**), PGXL ( **E**), KSR 2iL ( **F**), KSR 2iL+0.5 μM aza ( **G**), and KSR 2iL 0.5 μM PD03 ( **H**), or N2B27 2iL ( **I**). Scale bars represent 100 μm.

Pre-iPSCs represent an intermediate phase of reprogramming, but can be converted to fully pluripotency using small molecules ^21^. Thus, we transferred our *Acomys* pre-iPSCs into replicate wells and applied a variety of culture conditions intended to encourage full reprogramming. One well was maintained in KSR LIF dox as a control ( Figure 2A), but we removed dox in all other wells as acquisition of bona fide pluripotency is dependent upon transgene-independent self-renewal ^28^.

No colonies emerged from the KSR LIF condition, and untransfected fibroblasts in the well overgrew ( Figure 2B). In FAX, which supports primed ESCs that represent the post-implantation epiblast ^29^, no colonies emerged, and fibroblasts again overgrew ( Figure 2C). We also tested two media conditions used to sustain naïve human PSCs: t2iL Gö XYaa ^30^ and PXGL ^31^. Most cells died, and no colonies emerged in either condition ( Figure 2D,E). In addition to being used to culture ground state mouse ESCs ^32^, 2iL conditions containing inhibitors of MEK/ERK and GSK3 signaling along with LIF can induce *Mus* pre-iPSCs to convert to full pluripotency ^21, 33^. We switched cells into KSR 2iL, KSR 2iL plus 0.5 μm 5-azacytidine, or KSR 2iL with a titrated amount of PD03, which has been shown to support naïve-like human ESCs ^34^. The addition of 2iL caused an initial wave of cell death, but after 10 days, dome-shaped colonies emerged in all three KSR 2iL conditions and there was no noticeable difference between the different conditions ( Figure 2F–H). However, these conditions did not appear to be selective against the fibroblasts, potentially due to the presence of KSR in the media or expression of SV40 LT, and these fibroblasts overgrew. We mechanically picked colonies into new wells, but they collapsed soon after picking.

We also used serum-free N2B27 supplemented with 2iL to facilitate the transition to pluripotency. N2B27 2iL was much more selective than KSR 2iL, and almost no fibroblasts survived in these conditions, making it easy to identify the large, tightly-packed, dome-shaped colonies that emerged ( Figure 2I). Given the lack of proliferating fibroblasts in the culture, we attempted to enzymatically passage these N2B27 2iL colonies to a new well; however, the passaged cells did not survive.

### Acomys iPS-like cells are transgene-dependent

**Figure 3.**
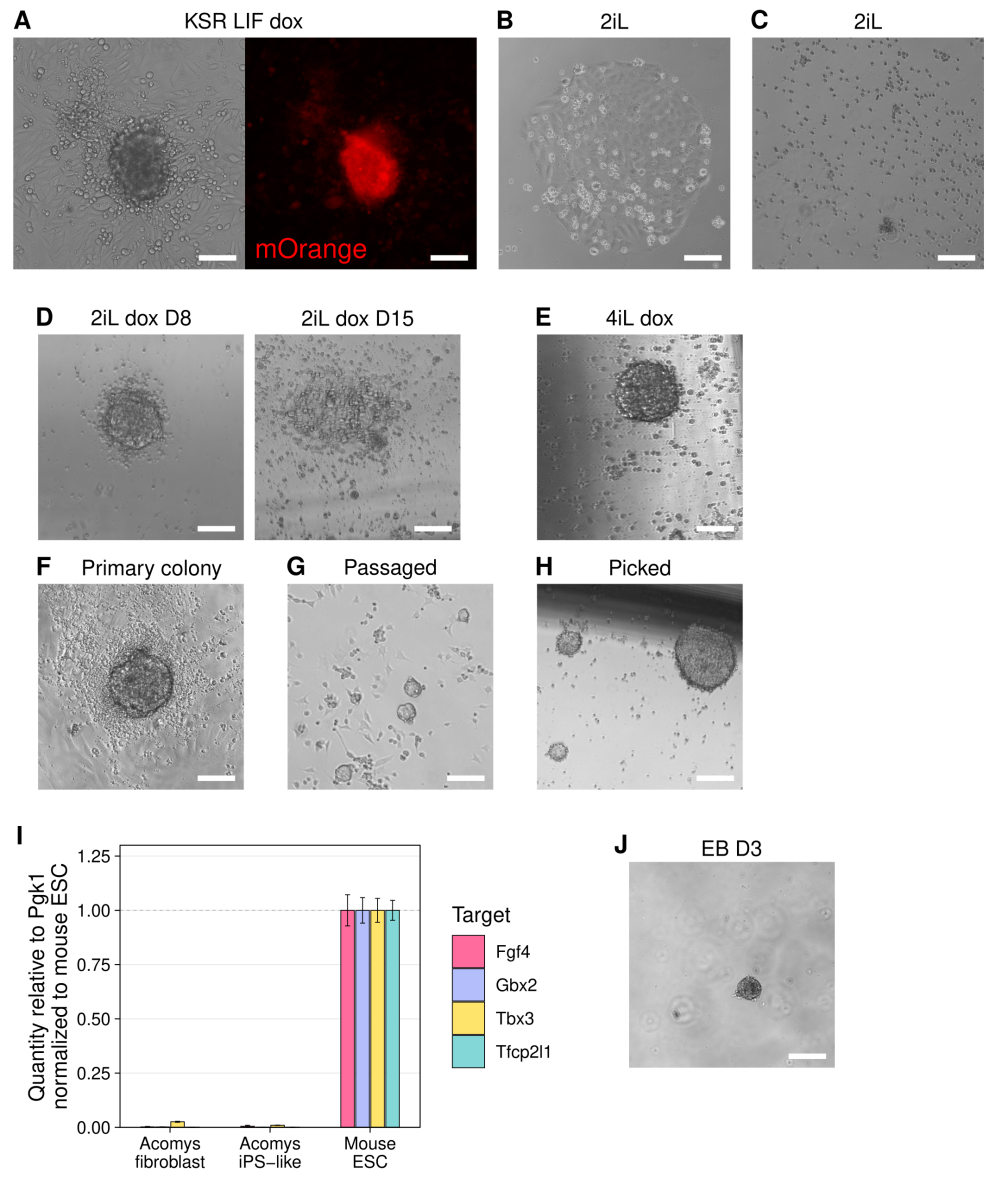
Transgene-dependent Acomys iPS-like cells resemble Mus PSCs but lack key features of pluripotency. **A**) Phase and mOrange images of colonies that arose from pre-iPSCs in KSR LIF dox. Scale bars represent 100 μm. **B**– **C**) Phase images of spontaneous endoderm-like differentiation ( **B**) and apoptosis ( **C**) after pre-iPSC colonies were picked into 2iL. Scale bars represent 100 μm. **D**) Phase images of an unstable iPS-like colony collapsing in 2iL dox followed from D8-D15. Scale bars represent 100 μm. **E**) Phase image of iPS-like colonies in 4iL dox. Scale bars represent 100 μm. **F**) Phase image of primary iPS-like colony in 4iL dox. Scale bar represents 100 μm. **G**– **H**) Phases image of iPS-like colonies in 4iL dox after enzymatic passaging ( **B**) or mechanical picking ( **C**). Scale bars represent 100 μm. **I**) RT-qPCR analysis of naïve pluripotency marker expression in *Acomys* iPS-like cells, *Acomys* fibroblasts, and *Mus* ESCs. Mean expression is shown relative to Pgk1 and normalized to *Mus* ESC level, ± SD (n=3 technical replicates). **J**) Phase image of necrotic mass of cells that remained after attempting hanging drop EB differentiation for 3 days using *Acomys* iPS-like cells. Scale bar represents 100 μm.

To alleviate the problems with fibroblast overgrowth observed in the more permissive culture conditions, we mechanically picked a colony of *Acomys* pre-iPSCs and expanded it in KSR LIF dox as a ‘pure’ population devoid of fibroblasts. After multiple passages, tightly-packed colonies of small cells spontaneously emerged ( Figure 3A). We mechanically picked these colonies, believing them to represent a more advanced state in the reprogramming process. These cells might potentially represent a delayed, stochastic path to iPS generation that avoids becoming trapped in the pre-iPS stage ^21^. However, when transferred to 2iL, these pre-iPSC colonies either differentiated to a primitive endoderm-like morphology or died ( Figure 3B,C).

Clearly, *Acomys* pre-iPSCs required sustained transgene induction to remain undifferentiated and survive. Thus, we transferred the aforementioned picked pre-iPSC colonies to 2iL dox and observed tightly packed, rounded colonies of *Acomys* iPS-like cells after 8 days. However, these iPS-like colonies exhibited cell death at their edges, and after 15 days, many colonies collapsed entirely ( Figure 3D). To support the remaining colonies we added Alk-5 inhibitor A83-01 and FGF receptor inhibitor PD173074, which are used to supplement 2iL to prevent differentiation of naked mole rat ^10^ and rat ^35^ iPSCs, respectively. This culture condition, termed 4iL dox, appeared to temporarily stabilize the iPS-like colonies ( Figure 3E). These *Acomys* iPS-like colonies survived passaging; however, they could not be maintained over multiple passages. Nevertheless, these experiments showed *Acomys* iPS-like cells could be derived from pre-iPSCs with sustained transgene induction.

We returned to a population of *Acomys* pre-iPSCs a single passage after induction, and after culturing these pre-iPSCs in 2iL dox conditions, tightly-packed, dome-shaped colonies emerged again. Shortly after, these colonies were switched to 4iL dox conditions ( Figure 3F). After enzymatic passaging, we observed small, rounded iPS-like colonies ( Figure 3G); however, several flatter pre-iPSCs remained and continued to proliferate, eventually overgrowing. When we instead mechanically picked primary iPS-like colonies into new wells, we obtained a pure population of iPS-like colonies devoid of flat pre-iPSCs ( Figure 3H). These iPS-like colonies had well-defined edges and were composed of cells with a high nucleus-to-cytoplasm ratio, characteristic of PSC colonies.

Surprisingly, however, these *Acomys* iPS-like cells did not exhibit upregulated naïve pluripotency marker expression ( Figure 3I). To determine whether these cells were functionally pluripotent despite not expressing expected pluripotency markers, we performed embryoid body (EB) differentiation. Though we attempted three different EB differentiation protocols using varying cell numbers, we were unable to obtain any differentiating EBs. In all attempts, the *Acomys* iPS-like cells aggregated but failed to proliferate and differentiate. Cell debris was observed in the media, and the aggregates appeared necrotic ( Figure 3J). The lack of pluripotency marker expression along with the failure to form EBs indicated these *Acomys* iPS-like cells, though morphologically similar to *Mus* PSCs, were not pluripotent.

### EOS-GiP system does not report pluripotent identity in Acomys

Since reliance upon morphological criteria to ascertain pluripotency of *Acomys* cells proved unsuccessful, we sought a fluorescent reporter to give a visual indication of pluripotency. We utilized a piggyBac EOS-GiP plasmid containing an EOS expression cassette driving expression of GFP and puromycin (puro) resistance. The EOS cassette is composed of a mouse early transposon (ETn) promoter, which is specific to PSCs, combined with Oct4- and Sox2-binding motifs found in PSC-specific enhancers ^36^. Thus, only PSCs should express GFP and survive puro treatment, allowing us to visually monitor and select for fully reprogrammed iPSCs.

**Figure 4.**
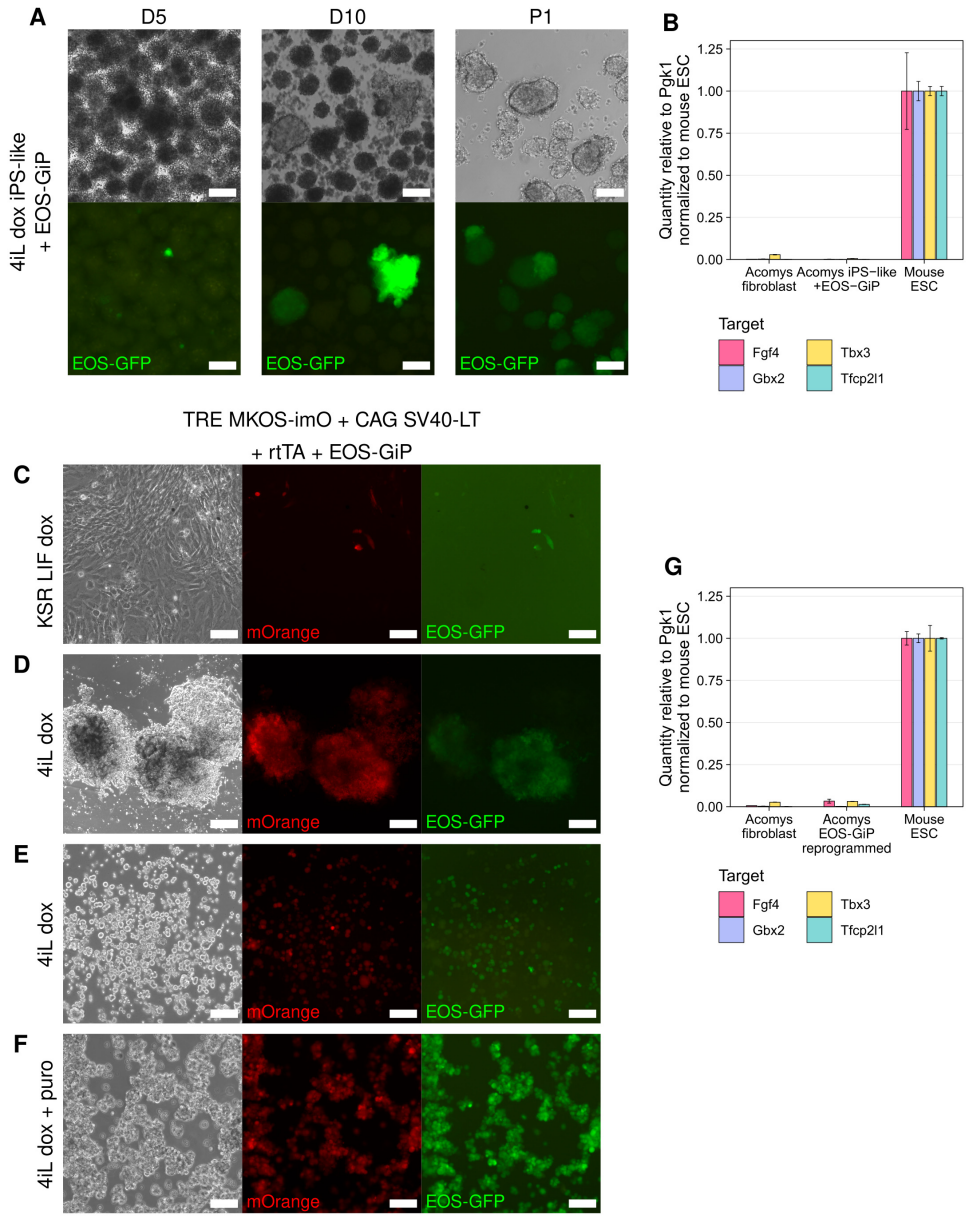
EOS-GiP does not provide a pluripotent identity readout in Acomys. **A**) Phase and GFP images of iPS-like cells in 4iL dox supplemented with 1 μg/ml puro at 5 (left) and 10 (center) days after transfecting with PB-EOS-GiP as well as after mechanical picking (right). Scale bars represent 100 μm. **B**) RT-qPCR analysis of naïve pluripotency marker expression in *Acomys* iPS-like cells with EOS-GiP knocked in, *Acomys* fibroblasts, and *Mus* ESCs. Mean expression is shown relative to Pgk1 and normalized to *Mus* ESC level, ± SD (n=3 technical replicates). **C**– **F**) Phase, mOrange, and GFP images of reprogramming fibroblasts in KSR LIF dox ( **A**), primary iPS-like colonies expressing EOS-GiP in 4iL dox ( **B**), pre-iPSCs expressing EOS-GiP in 4iL dox ( **C**), and cells expressing EOS-GiP in 4iL dox supplemented with 1 μg/ml puro ( **D**). Scale bars represent 100 μm. **G**) RT-qPCR analysis of naïve pluripotency marker expression in *Acomys* EOS-GiP cells, *Acomys* fibroblasts, and *Mus* ESCs. Mean expression is shown relative to Pgk1 and normalized to *Mus* ESC level, ± SD (n=3 technical replicates).

We first knocked EOS-GiP into previously generated *Acomys* iPS-like cells, conjecturing that there might exist a small population of iPSCs hidden among a majority of non-pluripotent cells. Following transfection, puro selection was applied. After 5 days, GFP-positive cells emerged, and after 10 days, GFP-positive colonies were picked and passaged. After a single passage, we had a pure population of GFP-positive *Acomys* iPS-like cells ( Figure 4A); however, these cells still did not express any of the pluripotency markers that we checked for ( Figure 4B). We hypothesized that the selective pressure from addition of puro immediately following introduction of the EOS-GiP construct led to selection for a population of cells containing aberrantly activated EOS-GiP, resulting in spurious GFP expression.

After performing transfections with EOS-GiP added to the reprogramming cocktail, we observed spurious GFP expression in transfected fibroblasts in KSR LIF dox ( Figure 4C). Past studies using a similar Oct4-GFP reporter system found reporter activation is not necessarily indicative of pluripotency in serum-containing media; however, transition of reprogramming intermediates to 2iL allowed them to progress to full pluripotency ^21^, and we would not expect to see spurious GFP expression in 2iL. We transitioned these EOS-GiP cells first to 2iL dox and then 4iL dox upon emergence of colonies. Indeed, select dome-shaped colonies were GFP-positive ( Figure 4D); however, some flat pre-iPSCs that clearly did not have an ES-like morphology also expressed GFP, suggesting that the EOS-GiP was not accurately reporting pluripotency ( Figure 4E). Nevertheless, we picked 24 dome-shaped colonies into separate wells and expanded them *.* Only one of the picked colonies remained GFP-positive so we passaged this colony and applied puro selection. The cells that survived selection had a distinctive morphology, growing in loose clumps of floating cells rather than in tightly packed, adherent colonies ( Figure 4F). Unsurprisingly, these EOS-GiP cells did not strongly express any of the pluripotency markers we assessed ( Figure 4G).

EOS-GiP does not provide a reliable readout of the pluripotent state in *Acomys* cells, though it is unclear why since this system has been used to track acquisition of pluripotency during reprogramming in several species including *Mus*, human, and even spiny rat ( *Tokudaia)* cells ^37, 38^. It is unlikely that sustained expression of reprogramming factors alone is driving EOS-GiP expression since similar dox-inducible reprogramming factors were utilized in *Tokudaia* without causing EOS-GiP misactivation ^37^. It is possible that there is some aspect of the *Acomys* transcriptional circuitry not present in other species causing this spurious activation; the mouse early transposon promoter may have broader activity in *Acomys*, or other transcription factors may have adapted to bind to the Oct4 or Sox2 binding motifs.

### Transgenic Nanog expression improves Acomys reprogramming

We next sought to test whether addition of Nanog to the reprogramming cocktail would facilitate complete reprogramming. Though dispensable during early stages of reprogramming, Nanog promotes the transition of pre-iPSCs to full naïve pluripotency ^39^. Furthermore, Nanog is only weakly or not expressed in partially reprogrammed cells that fail to fully activate the naïve pluripotency transcriptional circuitry ^1, 21^. Though the requirement for endogenous Nanog is system-dependent, Nanog overexpression still increases reprogramming efficiency in other systems utilizing the MKOS reprogramming cassette we are employing ^22^.

**Figure 5.**
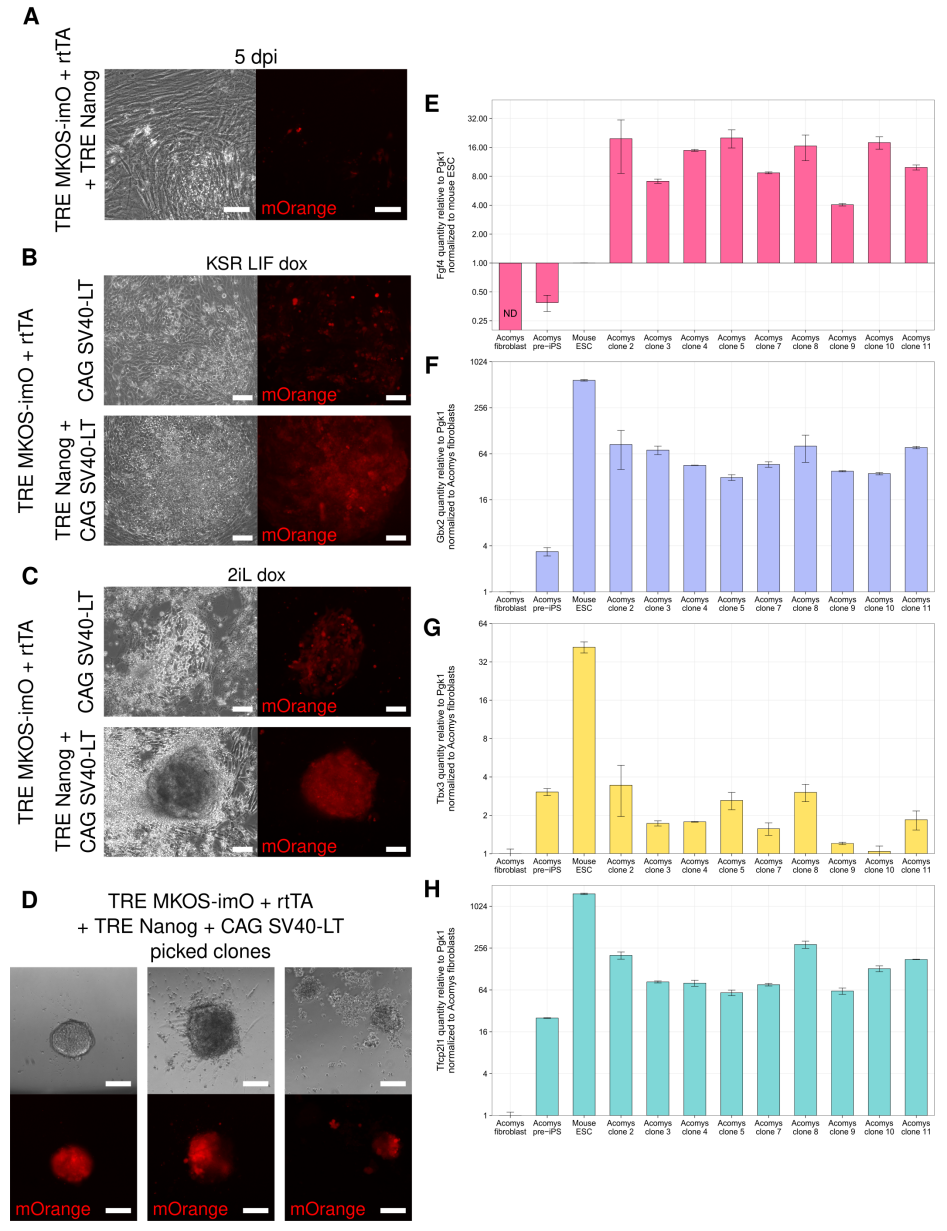
Nanog improves morphology and pluripotency marker expression in Acomys iPS-like cells. **A**) Phase and mOrange images of reprogramming fibroblast apoptosis in KSR LIF dox if SV40 LT is replaced with iNanog in the reprogramming cocktail. Scale bars represent 100 μm. **B**– **C**) Phase and mOrange images of pre-iPSCs expressing either SV40 LT alone or iNanog and SV40 LT in either KSR LIF dox ( **B**) or 2iL dox ( **C**). Scale bars represent 100 μm. **D**) Phase and mOrange images demonstrating the range of morphologies observed among picked iPS-like clones expressing iNanog and SV40LT. Scale bars represent 100 μm. **E**– **H**) RT-qPCR analysis of Tbx3 ( **E**), Gbx2 ( **F**), Tfcp2l1 ( **G**), and Fgf4 ( **H**) Log _2_ expression in clonal lines of iNanog *Acomys* iPSC-like cells, *Acomys* pre-iPSCs, *Acomys* fibroblasts, and *Mus* ESCs. Mean expression is shown relative to Pgk1 and normalized to *Acomys* fibroblast level ( **E**– **G**) or *Mus* ES level ( **H**), ± SD (n=3 technical replicates). Aco iN Cl2 signifies iNanog Acomys iPS-like cell Clone #2. Cl1 and Cl6 were excluded due to low cell numbers.

To overexpress Nanog, we integrated a piggyBac plasmid containing Nanog downstream of a dox-inducible TetO promoter into our reprogramming cocktail. Substitution of SV40 LT with Nanog in the reprogramming cocktail was insufficient to prevent widespread apoptosis and senescence ( Figure 5A). We then attempted to use SV40 LT, MKOS-imO, and Nanog in combination to reprogram *Acomys* fibroblasts. Nanog overexpression works synergistically with the DNA methyltransferase inhibitor 5-azacytidine (5-aza) to promote the final stages of reprogramming in pre-iPSCs ^33^, so at 11 dpi, we added 1 μM 5-aza to the media.

Nanog overexpression had a noticeable effect on the morphology of early reprogramming intermediates. *Acomys* pre-iPSCs without Nanog formed looser colonies composed of larger cells, with heterogeneous levels of mOrange. In contrast, *Acomys* pre-iPSCs expressing transgenic Nanog (iNanog) formed colonies with defined edges composed of very small, tightly packed cells ( Figure 5B) with more consistent mOrange expression. At 15 dpi, we switched the *Acomys* cells to 2iL dox with 1 μM 5-aza. iNanog *Acomys* pre-iPSCs formed mostly tightly packed, dome-shaped colonies composed of small cells, whereas pre-iPSCs without Nanog formed many looser colonies composed of larger cells ( Figure 5C).

In past experiments, we passaged cells in bulk or picked reprogramming colonies then pooled them together. In *Mus* and human contexts, properly reprogrammed cells outcompete non-reprogrammed cells which eventually senesce, so it is not necessary to pick and characterize individual colonies ^40^. However, since our non-reprogrammed cells could be immortalized by the SV40 LT and therefore remain in culture indefinitely, we picked 24 iNanog *Acomys* iPS-like colonies and cultured them as separate clonal lines. After picking, we observed that these lines exhibited a range of morphologies ( Figure 5D).

Only 11 of the iNanog clones survived mechanical passaging, of which 9 were successfully expanded for RT-qPCR analysis. *Acomys* iNanog iPS-like cell Tbx3 levels were slightly upregulated compared to *Acomys* fibroblast levels and similar to *Acomys* pre-iPS levels ( Figure 5E). Remarkably, levels of Gbx2, Tfcp2l1, and Fgf4 were all highly upregulated in *Acomys* iNanog iPS-like cells compared to both pre-iPSCs and fibroblasts, and these dramatic increases were consistent across all 9 clones assayed ( Figure 5F–H). *Mus* ESCs, used as a positive control for these RT-qPCR reactions, appeared to show far higher relative expression of Tbx3, Gbx2 and Tfcp2l1; however, it should be noted that direct cross-species comparisons are difficult to interpret as we do not know the absolute level of expression of these factors, or the housekeeping gene being normalized to, and they may differ significantly between *Acomys* and *Mus* naïve cells. Nevertheless, inclusion of Nanog in the reprogramming cocktail clearly induces upregulation of several naïve pluripotency markers that were not strongly expressed following reprogramming with the Yamanaka factors alone or in combination with SV40 LT in *Acomys.* Future work will elucidate whether these iNanog *Acomys* iPS-like cells are functionally pluripotent through differentiation and chimera assays.

## Discussion

Our data show traditional reprogramming protocols developed in *Mus* cannot be directly applied to *Acomys.* Nevertheless, this preliminary work provides several avenues for future investigation.

The requirement for SV40 LT during reprogramming suggests a hyperactive tumor suppressor response in *Acomys*. Immortalization increases reprogramming efficiency in *Mus* and human ^41^ and also greatly enhances reprogramming in *Heterocephalus* ^10, 12^. Tan *et al.* found *Heterocephalus* cells require SV40 LT to undergo reprogramming, mirroring our findings in *Acomys* ^12^. Lee *et al.* independently found adult *Heterocephalus* fibroblasts could be reprogrammed without SV40 LT; however, colonies emerged at day 43, which was longer than we cultured our *Acomys* cells ^10^. It is possible *Acomys* reprogramming requires more time, though this is unlikely given the extensive apoptosis and senescence we observed relatively early compared to these timescales.

*Heterocephalus* has a stable epigenome that resists dedifferentiation, characterized by histones marked more by H3K27me3 repressive marks than H3K4me3 activating marks, and expression of SV40 LT opened previously closed reprogramming factor promoters ^12^ *.* Since the epigenetic landscape is reset to facilitate reprogramming ^42^, it is possible *Acomys* possesses a similarly stable epigenome. Past studies showed *Acomys* skin exhibits resistance to UV radiation-induced DNA damage and age-related senescence, drawing further parallels with *Heterocephalus,* a model of cancer resistance and longevity ^43^. This suggests that more extensive epigenetic remodeling may be required to fully revert Acomys cells to a pluripotent identity. *Acomys* could possibly be resistant to tumorigenesis, similar to *Heterocephalus* and the regenerative axolotl salamander ^44^, warranting further study into cancer in *Acomys.*

It is possible the non-pluripotent *Acomys* iPS-like cells we generated without Nanog represented transformed cells akin to cancer stem cells as many of the same mechanisms control reprogramming and oncogenesis ^45^. By blocking p53 and Rb tumor suppressors, SV40 LT enhances reprogramming; however, it can also play a role in cancer initiation ^46^. Mali *et al.* used SV40 LT to generate human iPSCs, resulting in two distinct types of colonies: bona fide iPSCs and nullipotent cells that were morphologically indistinguishable. The nullipotent cells were not positive for certain pluripotency markers and failed to form EBs ^47^, similar to our *Acomys* iPS-like cells without transgenic Nanog. Despite this drawback, SV40 LT was necessary to generate *Acomys* reprogramming intermediates. Many parallels exist between cancer and regeneration ^48^, and tumor suppressors play a key role in preventing tumorigenesis during axolotl salamander and zebrafish regeneration ^49, 50^. Thus, it will be of interest to characterize the role of tumor suppressors during *Acomys* regeneration which, like reprogramming and cancer, involves undifferentiated, proliferative cells ^51^.

In order to improve the reprogramming of *Acomys* cells, several considerations should be made. A better understanding of *Acomys* reproductive biology would give a point of reference to guide reprogramming efforts. Although a transcriptome exists for gene expression during the earliest stages of embryonic development ^19^, a thorough understanding of the gene expression network in the pre-implantation naïve epiblast will be important for a more comprehensive characterization of putative *Acomys* iPSCs. Differences between *Mus* and *Acomys* reproductive biology make it difficult to assume development occurs similarly. Significant differences exist in the hormones needed to stimulate superovulation as well as the timing of ovulation in *Acomys* ^52^. Strikingly, *Acomys* is the only known rodent capable of menstruation ^53^, and embryonic genome activation in *Acomys* is more human-like than that of *Mus* ^19^ *.* Continued study of the *Acomys* reproduction and development will inform efforts to improve iPSC generation.

The choice of starting cell can also have a significant impact on reprogramming. We used *Acomys* neonatal fibroblasts due to their ease of acquisition; however, it has been shown that human postnatal fibroblasts exhibit lower reprogramming efficiency compared to embryonic fibroblasts ^26^. Unfortunately, acquiring embryonic starting materials is difficult since *Acomys* embryonic development is not well characterized. Furthermore, somatic stem cells reprogram more efficiently than differentiated cells, potentially because they do not express as many lineage specific genes, which inhibit reprogramming ^54^. Thus, it may be of interest to derive tissue-specific stem cells to be used as a starting material in the future.

Given the deceptive appearance of non-pluripotent *Acomys* iPS-like cells, morphology cannot be used as a reliable indicator of pluripotency. Thus, we sought to use an EOS-GiP reporter to monitor achievement of pluripotency *in vitro;* however, we found widespread spurious activation in *Acomys* cells. This exogenous reporter integrates randomly in the genome; however, an endogenous reporter would present a more accurate reflection of gene regulation since it is placed within the appropriate chromatin context ^55^. Unfortunately, without an annotated genome, it would be extremely difficult to develop an *Acomys* endogenous pluripotency reporter line, further highlighting difficulties in working with this non-traditional model organism.

There remains a possibility that additional factors may be required to induce pluripotency in *Acomys.* For instance, Lin28 increases the kinetics of reprogramming in a cell proliferation-dependent manner, similar to the effects of p53 knockdown ^56^. Furthermore, iPSCs have been successfully generated using a cocktail combining the Yamanaka factors with Nanog and Lin28 in several species ^57^. Thus, future experiments adding Lin28 or other factors to the reprogramming cocktail might enhance reprogramming further.

Generation of transgene-dependent *Acomys* iPSCs would subsequently allow for the screening of chemical compounds to determine the species *-*specific culture conditions necessary to maintain *Acomys* PSCs, independent of exogenous transgene expression. A similar approach was previously used to identify the culture conditions supportive of the human naïve state ^58^. Signaling requirements for pluripotency maintenance vary from species to species ^59^, but *Rattus, Heterocephalus,* and *Tokudaia* iPSCs can be cultured transgene-free in 2iL conditions with only slight modifications ^10, 37, 60^, suggesting the same may hold true for *Acomys.*

The work presented here identifies *Acomys-*specific obstacles to reprogramming and provides the preliminary work necessary to successfully reprogram *Acomys* cells. The requirement for SV40 LT during initial dedifferentiation of *Acomys* fibroblasts suggests tumor suppressor mechanisms might tightly control cell identity change during *Acomys* regeneration. We also showed that overexpression of Nanog induces upregulation of several pluripotency markers in *Acomys* iPS-like cells. In summary, there are several avenues of exploration that could potentially lead to improved generation of *Acomys* iPSCs.

If successful, bona fide *Acomys* iPSCs would allow for the development of transgenic animals, chimeras, and organoid models *,* all of which would contribute greatly to our understanding of *Acomys* regeneration. Overall, continued study of this emerging, non-traditional model organism could have broad implications in the fields of wound healing, oncology, and cellular plasticity.

## Data availability

### Underlying data

Open Science Framework: Tumor suppressors inhibit reprogramming of African spiny mouse, DOI. https://doi.org/10.17605/OSF.IO/VWKYT ^61^.

This project contains the following underlying data:

- Uncropped and unedited image files for Figures 1-5
- Uncropped adjusted image files for mCherry for Figures 1-5
- qPCR data for Figures 1-5

Data are available under the terms of the Creative Commons Zero "No rights reserved" data waiver (CC0 1.0 Public domain dedication).
