# Supplementary material for: Tumor suppressors inhibit reprogramming of African spiny mouse ( Acomys) fibroblasts to induced pluripotent stem cells
Source: Wellcome Open Res. 2022 Aug 18;7:215. [Version 1] doi: 10.12688/wellcomeopenres.18034.1 (PMC9437536; doi:10.12688/wellcomeopenres.18034.1)
Supplement: Peer review history

# Peer review: Reviewer response for version 1

**DOI:** 10.21956/wellcomeopenres.19998.r52101

**Published:** 2022-09-01

**Authors:** Jianlong Wang

**Affiliations:** 1 Department of Medicine, Columbia Center for Human Development and Stem Cell Therapies, Columbia Stem Cell Initiative, Herbert Irving Comprehensive Cancer Center, Columbia University Irving Medical Center, New York, NY, USA

The African spiny mouse ( *Acomys*) is an emerging mammalian model for scar-free regeneration, and further study of *Acomys *could advance the field of regenerative medicine. The work presented by Dr. Silva and their colleagues identifies *Acomys*-specific obstacles to reprogramming and provides the preliminary work necessary to reprogram *Acomys* cells successfully. The authors found that SV40 LT is required during initial dedifferentiation of *Acomys* fibroblasts upon classic Yamanaka MKOS-mediated reprogramming, which brought up an interesting point suggesting tumor suppressor mechanisms might tightly control cell identity change during *Acomys* regeneration. The authors also showed that overexpression of Nanog induces upregulation of several pluripotency markers in *Acomys* iPS-like cells. While the final outcome and the status of bona fide *Acomys* iPSC are far from satisfactory at the moment, the current study does provide several avenues of exploration that could potentially lead to the improved generation of *Acomys* iPSCs. The multiple trials and painstaking troubleshooting efforts the authors put into this study are highly appreciated by this reviewer. Continued study of this emerging, non-traditional model organism could have broad implications in wound healing, oncology, and cellular plasticity. If successful, bona fide *Acomys* iPSCs would allow for the development of transgenic animals, chimeras, and organoid models, all of which would contribute significantly to our understanding of *Acomys* regeneration.

 One potential weakness of this study is the lack of a “last kick in front of the door to score the final goal” when the authors state, “Future work will elucidate whether these iNanog Acomys iPS-like cells are functionally pluripotent through differentiation and chimera assays.” While chimera assays are time-consuming, I am eager to know whether these iNanog *Acomys* iPS-like cells are functionally pluripotent through EB differentiation, which should be relatively quick and straightforward to perform. However, this is certainly something on the authors’ agenda and could be part of their next report.  

Is the work clearly and accurately presented and does it cite the current literature?

Yes

If applicable, is the statistical analysis and its interpretation appropriate?

Yes

Are all the source data underlying the results available to ensure full reproducibility?

Yes

Is the study design appropriate and is the work technically sound?

Yes

Are the conclusions drawn adequately supported by the results?

Yes

Are sufficient details of methods and analysis provided to allow replication by others?

Yes

Reviewer Expertise:

Stem cell pluripotency and somatic cell reprogramming; stemness control in pluripotent and cancer cells; gene expression and regulation;

I confirm that I have read this submission and believe that I have an appropriate level of expertise to confirm that it is of an acceptable scientific standard.

# Peer review: Reviewer response for version 1

**DOI:** 10.21956/wellcomeopenres.19998.r52098

**Published:** 2022-08-26

**Authors:** Keisuke Kaji

**Affiliations:** 1 Centre for Regenerative Medicine, Institute for Regeneration and Repair, University of Edinburgh, Edinburgh, UK

In this manuscript, the authors attempted to generate iPSCs from the African spiny mouse (Acomys) fibroblasts. Expression of only Oct4, Sox2, Klf4, cMyc (OSKM) resulted in no proliferating cells due to apoptosis and senescence. Addition of SV40 large T antigen (SV40 LT), as well as the use of 2i (CHIR99021 + PD0325901) and LIF (2iL), allowed proliferation of cells with iPSC-like morphology in the presence of OSKM, but they did not have pluripotency gene expression and were not able to differentiate. Finally, the use of SV40 LT and Nanog together with OSKM in the presence of 2iL and 5-azacytidine allowed them to obtain iPSC-like cells with Fgf4, Gbx2, Tfcp2l1 expression. Expression of other pluripotency genes and differentiation capacity are to be determined.

 As the authors stated, Acomys iPSCs would be a really interesting research tool to understand regeneration capacity of Acomys. Particularly the generation of chimeric mice would be exciting. As shown with rat ESCs, mice with specific Acomys organ could also be generated by using tissue specific master transcription factors knockout mouse embryos as a recipient embryos for chimera.  

 This work has demonstrated that the generation of Acomys iPSCs is not straightforward, but probably possible with further optimization. Up-regulation of some of pluripotency genes is promising, while deeper characterization, and perhaps more optimization, would be needed. If the cell lines presented here are pluripotent, they could be a useful tool to identify optimal culture condition, even if they are exogenous factor dependent, as the authors described.

 As a minor point, the definition of "pre-iPSCs" were not clear to me. What is the difference between pre-iPSCs and iPSC-like cells without pluripotentcy? I believe there is no clear definition in the research field, but some definitions within this manuscript could have been useful for the readers.

Is the work clearly and accurately presented and does it cite the current literature?

Yes

If applicable, is the statistical analysis and its interpretation appropriate?

Yes

Are all the source data underlying the results available to ensure full reproducibility?

Yes

Is the study design appropriate and is the work technically sound?

Yes

Are the conclusions drawn adequately supported by the results?

Yes

Are sufficient details of methods and analysis provided to allow replication by others?

Yes

Reviewer Expertise:

Reprogramming, pluripotency

I confirm that I have read this submission and believe that I have an appropriate level of expertise to confirm that it is of an acceptable scientific standard.
